# 1887. Hyperglycemia and COVID-19: An independent risk factor for disease severity and death in the Dominican Republic

**DOI:** 10.1093/ofid/ofac492.1514

**Published:** 2022-12-15

**Authors:** Yori A Roque, David De Luna, Alfredo J Mena Lora, Lisa Maria Castillo Mercado, Carlos Perdomo, Mariela Acosta

**Affiliations:** Hospital Metropolitano de Santiago (HOMS), Santiago, Santiago, Dominican Republic; Hospital Metropolitano de Santiago, Santiago, Santiago, Dominican Republic; University of Illinois Chicago, Chicago, Illinois; Hospital Regional Universitario Jose Maria Cabral y Baez, Santiago, Santiago, Dominican Republic; Hospital Regional Universitario José Maria Cabral y Baez, Santiago de los caballeros, Santiago, Dominican Republic; Hospital Regional Universitario Jose Maria Cabral y Baez, Santiago, Santiago, Dominican Republic

## Abstract

**Background:**

The COVID-19 pandemic has caused an unprecedented global public health emergency. Vaccine uptake in low and middle income countries (LMICs) lags developing nations and immunity from vector-based vaccines commonly used in LMICs may be inferior to mRNA vaccines. Thus, defining clinical characteristics that can help identify and triage cases and allocate resources in LMICs of priority. Hyperglycemia has been associated with higher morbidity and mortality in numerous diseases and in critical illness. We seek to understand the relationship between COVID-19 and hyperglycemia.

**Methods:**

This is a single center retrospective review of cases with COVID-19 between January 2021 and June 2021. Adult patients >18 years of age were reviewed and those with a molecular-based laboratory confirmed SARS-CoV-2 infection were included in our study. Patients with known diabetes, elevated A1C or prior steroid use within 2 weeks of admission were excluded. Clinical characteristics, demographics, glucose levels, C-reactive protein (CRP) and ferritin were reviewed.

**Results:**

A total of 120 patients were reviewed, of which 60.8% were male. Hyperglycemia ( >140mg/dL) was present in 57.5%. Hyperglycemia was associated with elevation of inflammatory markers including CRP and Ferritin (p=0.12) (Table 1). Hyperglycemia was more common in patients requiring supplemental low flow oxygen (table 2) and was more common in patients who did not survive (Figure 1). The mortality rate was higher in the hyperglycemia group with 61.5%, a statistically significant finding.

Association between hyperglycemia and inflammatory markers.

**Figure:**
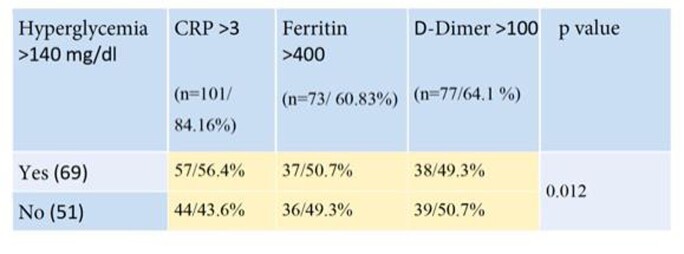

Supplemental oxygen needs and hyperglycemia.

**Figure:**
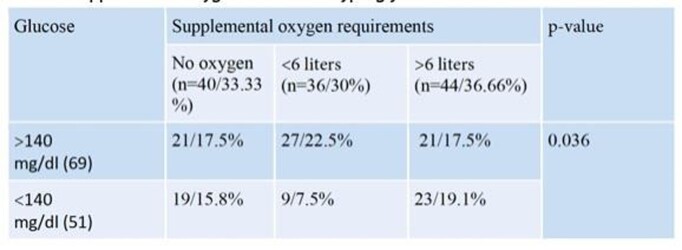

Survival and hyperglycemia.

**Figure:**
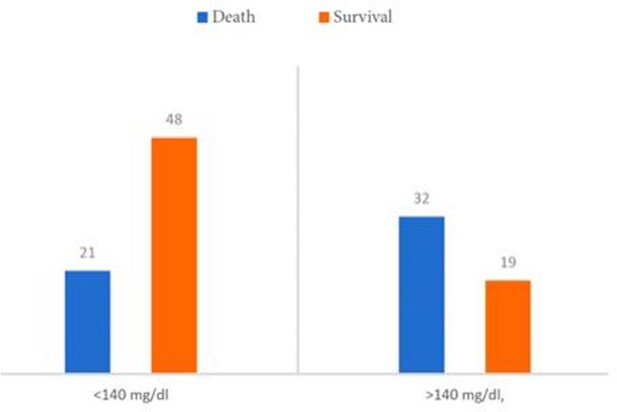

**Conclusion:**

Hyperglycemia on admission was an independent risk factor for disease progression and death. Inflammatory markers were also higher in patients with hyperglycemia. These patients had no prior steroid use or diabetes. Thus, it is possible that it reflects inflammation, stress, or endocrine end-organ damage due to SARS-CoV-2. If validated in larger studies, this simple test can help clinicians identify patients at risk of decompensation and allocate resources and therapeutics accordingly.

**Disclosures:**

**All Authors**: No reported disclosures.

